# P-704. RSV Signs and Symptoms at Time of Hospitalization among Pediatric and Adult Population with RSV in two Seasons: A Real-World Data Analysis using Electronic Health Records in the US

**DOI:** 10.1093/ofid/ofaf695.916

**Published:** 2026-01-11

**Authors:** Sima S Toussi, Negar Niki Alami, Edward Weinstein, Lulu Lee, Stacey Purinton, Vicky W Li, Robert J Taylor, Neelanzana Paudel, Wing Yu Tang

**Affiliations:** Pfizer Inc., New York, New York; Pfizer Inc., New York, New York; Pfizer Inc., New York, New York; Oracle Life Sciences, Austin, Texas; Oracle Life Sciences, Austin, Texas; Oracle Life Sciences, Austin, Texas; Oracle Life Sciences, Austin, Texas; Oracle Life Sciences, Austin, Texas; Pfizer Inc., New York, New York

## Abstract

**Background:**

Respiratory syncytial virus (RSV) is a common respiratory illness, with certain populations at higher risk of severe disease including pediatric patients, older adults, and those with compromised immune systems. The current analyses aim to examine the signs and symptoms (S&S) of RSV at time of hospitalization.

**Figure:**
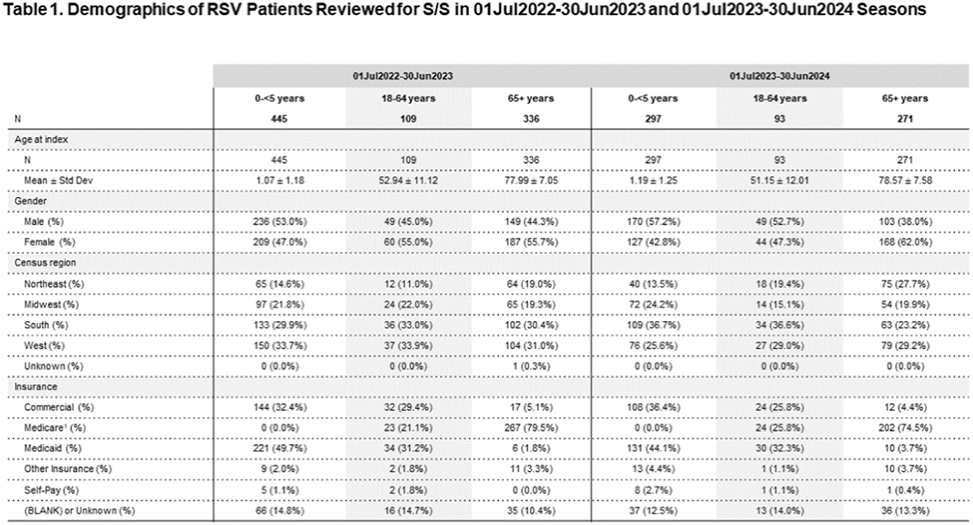

**Figure:**
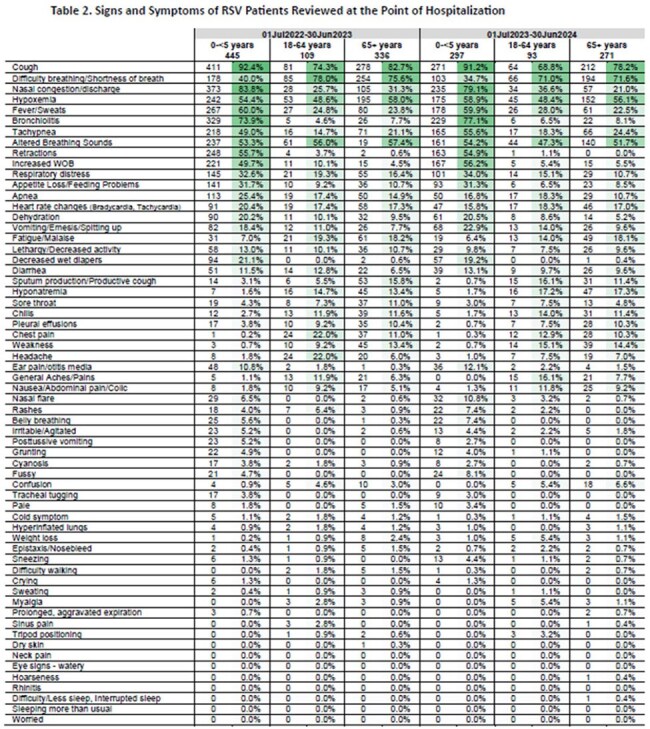

**Methods:**

A retrospective analysis was conducted using structured electronic health records (EHR) data from Oracle Life Sciences’ nationally representative EHR database. Patients hospitalized with RSV (identified via ICD-9, ICD-10, and/or SNOMED-CT codes) within the two seasons of interest (Season 1 [S1]: 2022Jul01-2023Jun30 and Season 2 [S2]: 2023Jul01-2024Jun30; qualifying once per season) were randomly selected for comprehensive review of S&S. RSV S&S included were on the basis of historic literature, S&S instruments, and expert opinion. Incidence of each S&S was determined via structured (e.g. ICD, SNOMED-CT) and/or manual physician notes review of each S&S text. Descriptive statistics of RSV S&S were reported by age stratification (pediatrics: 0< 5; at-risk adults: 18-64, 65+).

**Results:**

A total of 890 and 661 patients were randomly selected from S1 and S2, respectively. Demographics are presented in Table 1 and S&S are presented in Table 2. Overall S&S incidence in children and adults had overlapping similarities, with common S&S (e.g. cough, altered breathing sounds, hypoxemia) found in all age groups. Certain S&S were higher among pediatric patients (e.g. nasal congestion, fever, appetite loss/feeding problems) while other S&S were higher in older adults (e.g. fatigue). Incidence patterns were similar between both seasons of the predominant S&S found across age groups.

**Conclusion:**

This real-world analysis of RSV patients revealed some overlapping similarities of RSV S&S present at the point of hospitalization. Further characterization of RSV signs & symptoms can help inform natural history of the disease and timely interventions.

**Disclosures:**

Sima S. Toussi, MD, Pfizer: Stocks/Bonds (Private Company) Negar Niki Alami, MD, Pfizer: Employee|Pfizer: Stocks/Bonds (Public Company) Edward Weinstein, MD, PhD, Pfizer: Employee|Pfizer: Stocks/Bonds (Public Company) Lulu Lee, PhD, Oracle Life Sciences: Employed by Oracle Life Sciences, which received funding from Pfizer to conduct the study. Stacey Purinton, MSN, MPH, Oracle Life Sciences: Employed by Oracle Life Sciences, which received funding from Pfizer to conduct the study. Vicky W. Li, MPH, Oracle Life Sciences: Employed by Oracle Life Sciences, which received funding from Pfizer to conduct the study. Robert J. Taylor, AS, Oracle Life Sciences: Employed by Oracle Life Sciences, which received funding from Pfizer to conduct the study. Neelanzana Paudel, MS, Oracle Life Sciences: Employed by Oracle Life Sciences, which received funding from Pfizer to conduct the study. Wing Yu Tang, MPH, Pfizer: Employee|Pfizer: Stocks/Bonds (Public Company)

